# Outcomes of Laparoscopic Hiatal Hernia Repair: A Three-Year Follow-Up Study From a UK Tertiary Centre

**DOI:** 10.7759/cureus.94404

**Published:** 2025-10-12

**Authors:** Muhammad U Khan, Amr Alnagar, Atiya Hameedulilah, Syed Osama Zohaib Ullah

**Affiliations:** 1 General Surgery, University Hospital Birmingham, Birmingham, GBR

**Keywords:** laparoscopic hiatal hernia repair, long-term outcomes, paraesophageal hernia, radiological recurrence, redo surgery, sliding hiatal hernia

## Abstract

Background: Laparoscopic hiatal hernia repair is standard for symptomatic para-oesophageal and selected sliding hernias. International guidance supports elective repair for symptomatic disease; however, reported radiological recurrence rates vary, and their clinical relevance is debated.

Methodology: Retrospective cohort of 126 adults undergoing elective laparoscopic hiatal hernia repair at University Hospitals Birmingham (2016-2020). All patients underwent standard laparoscopic reduction, posterior cruroplasty and Nissen (360°) fundoplication. No mesh reinforcement was used in any case. Follow-up at 12 and 36 months captured overall symptom relief, dysphagia improvement, radiological recurrence, redo surgery, and symptomatic patients without radiological recurrence. Outcomes are intention-to-follow (*N* = 126) and reported as *n* (%) with 95% confidence intervals (CIs).

Results: The median age was 59 years. At 12 months, 111/126 (88.1%) reported overall symptom relief, and 98/126 (77.8%) reported dysphagia improvement. At 36 months, 103/126 (81.7%) and 88/126 (69.8%), respectively. Radiological recurrence occurred in 15/126 (12.0%) at 12 months and 28/126 (22.2%) at 36 months. Symptomatic recurrence requiring redo surgery occurred in 2/126 patients (1.6%) at 12 months and 9/126 (7.1%) at 36 months; asymptomatic recurrences were 13/126 (10.3%) and 19/126 (15.1%), respectively. Symptomatic patients without radiological recurrence were 3/126 (2.4%) at 12 months and 4/126 (3.2%) at 36 months.

Conclusions: Laparoscopic repair provides a reliable symptom control outcome through three years. Radiological recurrence is relatively common but frequently asymptomatic; only a minority (≤10%) require redo surgery. Long-term, symptom-led follow-up is warranted.

## Introduction

Hiatal hernia is associated with gastroesophageal reflux, dysphagia, and, rarely, acute volvulus. Minimally invasive repair has largely replaced open approaches, offering faster recovery and lower perioperative risk. Guidance from the Society of American Gastrointestinal and Endoscopic Surgeons (SAGES) supports operative repair for symptomatic para-oesophageal hernias and emphasises shared decision-making around mesh use and fundoplication [1]. UK service guidance also highlights the importance of managing these patients within specialist upper gastrointestinal units [2].

Despite technical standardisation, the long-term durability of repair remains debated. Large series and meta-analyses report radiological recurrence rates of approximately 15-35% at mid- to long-term follow-up, but redo surgery is required far less frequently, and quality of life typically remains improved [3-5]. Recurrence risk is influenced by both patient and technical factors, and many recurrences are small and clinically insignificant [3,4].

This study aimed to evaluate the three-year outcomes of laparoscopic hiatal hernia repair at a UK tertiary centre, with particular focus on the durability of symptom relief and the clinical significance of anatomical recurrence. It examined the rates of radiological recurrence, the incidence of clinically significant recurrence necessitating redo surgery, and the persistence of symptoms in patients without anatomical recurrence, thereby providing a comprehensive assessment of both anatomical and symptomatic outcomes over the three-year follow-up period.

## Materials and methods

This was a retrospective cohort study conducted at University Hospitals Birmingham and included consecutive adults who underwent elective laparoscopic hiatal hernia repair between January 2016 and December 2020.

Adults aged 18 years and older with symptomatic hiatal hernia confirmed on endoscopy and/or radiology who underwent primary laparoscopic repair were included. Exclusion criteria were paediatric cases, incidental or asymptomatic hernias, open or revisional repairs, concomitant oesophagogastric resections, and incomplete follow-up.

All patients underwent standard laparoscopic reduction, posterior cruroplasty, and Nissen’s fundoplication. No mesh reinforcement was used. Procedures were performed within the same department by three consultant surgeons of comparable experience and competency in upper gastrointestinal surgery. Details such as the use of mesh were not analysed in this audit and are therefore not reported.

Follow-up at 12 and 36 months was used to assess patient-reported outcomes, including overall symptom relief and improvement in dysphagia; anatomical recurrence was defined as any recurrence identified on barium swallow, CT, or endoscopic evaluation. Endoscopic recurrence referred to visible hiatal or wrap disruption confirmed at upper endoscopy. The need for redo surgery was recorded for symptomatic recurrence, and persistent or recurrent symptoms without anatomical recurrence were also documented.

Analyses followed an intention-to-follow approach for the entire cohort of 126 patients. Categorical outcomes are presented as absolute numbers and percentages with 95% Wilson confidence intervals. Point estimates were rounded to whole patients where applicable. Given the descriptive design, no hypothesis testing was undertaken.

## Results

A total of 126 patients underwent laparoscopic hiatal hernia repair, with a median age of 59 years. The demographic and baseline characteristics of the cohort are summarised in Table 1.

**Table 1:** Patient demographics (N = 126). The table summarises baseline demographic and clinical characteristics, including comorbidities. Values are descriptive only; no comparative statistical analysis was performed. COPD, chronic obstructive pulmonary disease

Characteristic	Value
Age (years), mean ± SD	58.2 ± 10.4
Sex	
Male	58 (46.0%)
Female	68 (54.0%)
Hernia type	
Sliding	76 (60.3%)
Para-oesophageal	50 (39.7%)
Comorbidities	
Hypertension	34 (27.0%)
Diabetes mellitus	29 (23.0%)
COPD	14 (11.1%)

**Figure 1:**
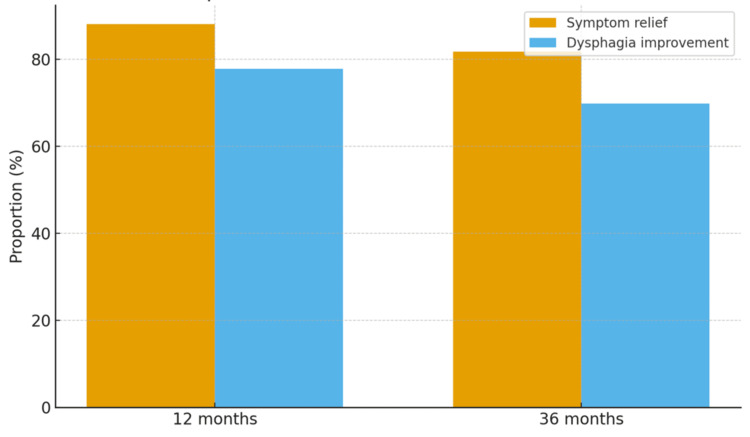
Patient-reported outcomes at 12 vs. 36 months (N = 126).

At the 12-month assessment, 111 patients (88.1%) reported overall symptom relief, and 98 patients (77.8%) experienced improvement in dysphagia. At 36 months, 103 patients (81.7%) continued to report overall symptom relief, while 88 patients (69.8%) reported sustained improvement in dysphagia. These findings demonstrate durable, though slightly reduced, symptom benefit over time. The distribution of patient-reported outcomes at both time points is shown in Figure 1.

**Figure 2:**
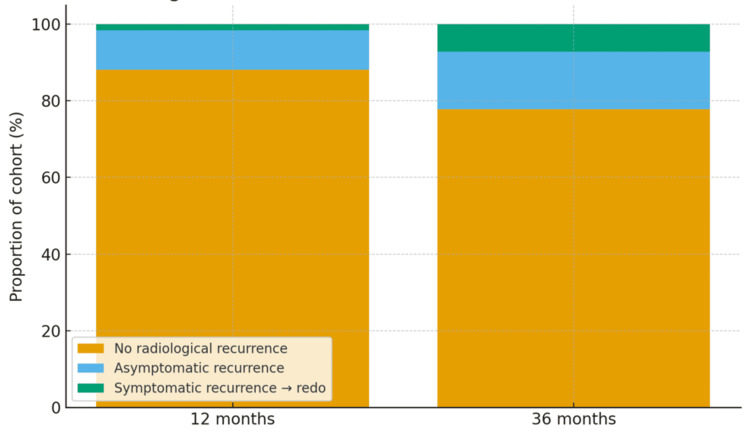
Radiological recurrence status at 12 vs. 36 months (N = 126).

Radiological recurrence was observed in 15 patients (12.0%) at 12 months and 28 patients (22.2%) at 36 months. Despite this increase, the majority of recurrences remained clinically silent. Only two patients (1.6%) required redo surgery at 12 months, increasing to nine patients (7.1%) at 36 months, while the remainder, 13 patients (10.3%) at 12 months and 19 patients (15.1%) at 36 months, were asymptomatic and managed conservatively. A small subgroup reported persistent symptoms despite the absence of radiological recurrence, affecting three patients (2.4%) at 12 months and four patients (3.2%) at 36 months. The breakdown of recurrence patterns and their clinical significance are illustrated in Figure 2 and detailed in Table 2.

**Table 2:** Clinical and radiological outcomes at 12 and 36 months (N = 126). The table presents clinical and radiological outcomes at 12 and 36 months following laparoscopic hiatal hernia repair. Outcomes include overall symptom relief, dysphagia improvement, radiological recurrence, and its subcategories (symptomatic recurrence requiring redo surgery, asymptomatic recurrence managed conservatively, and symptomatic patients without radiological recurrence). Statistical comparisons between 12- and 36-month outcomes were performed using the chi-square test. Both the test statistic (χ²) and the corresponding *P*-value are reported. A *P*-value <0.05 was considered statistically significant.

Outcome	12 months	36 months	Test statistics	*P*-value
Symptom relief	111 (88.1%)	103 (81.7%)	*χ*² = 1.52	0.218
Dysphagia improvement	98 (77.8%)	88 (69.8%)	*χ*² = 1.66	0.197
Radiological recurrence	15 (12.0%)	28 (22.2%)	*χ*² = 4.04	0.044*
Symptomatic recurrence → redo surgery	0 (0%)	9 (7.1%)	*χ*² = 7.37	0.007**
Asymptomatic recurrence (no surgery)	15 (12.0%)	19 (15.1%)	*χ*² = 0.42	0.515
Symptomatic without radiological recurrence	3 (2.4%)	6 (4.8%)	*χ*² = 0.76	0.383

Overall, these outcomes indicate that while radiological recurrence becomes more common with time, the majority of recurrences remain clinically silent. Importantly, durable symptom relief is maintained in over four-fifths of patients at three years, and fewer than one in 10 require a reoperation. This highlights the distinction between anatomical recurrence and clinically relevant failure, emphasising that radiological findings alone should not drive surgical decision-making.

## Discussion

In this tertiary-centre cohort, laparoscopic hiatal hernia repair provided durable symptom control over a three-year follow-up period. More than four-fifths of patients continued to report overall symptom relief at 36 months, and nearly 70% experienced sustained improvement in dysphagia. These findings highlight the effectiveness of laparoscopic repair in delivering long-term clinical benefit, although some attenuation of symptom control was observed over time.

Our findings further demonstrate that, although radiological recurrence becomes more frequent with time, the majority of recurrences remain clinically silent and do not necessitate surgical intervention. Importantly, reliable symptom relief was maintained in over four-fifths of patients at three years, with fewer than one in 10 requiring redo surgery. This distinction between anatomical recurrence and clinically relevant failure underscores the need to interpret radiological findings within the context of patient symptoms and overall functional outcomes. In clinical practice, this suggests that routine radiological surveillance may overestimate failure rates and that management decisions should be guided primarily by symptom recurrence and patient-reported quality of life. These results align with an increasingly symptom-focused approach advocated by contemporary guidelines and long-term studies, which emphasise the importance of patient-centred follow-up rather than radiological monitoring alone.

Radiological recurrence increased between the first and third year of follow-up, rising from 12.0% to 22.2%. However, the majority of recurrences were asymptomatic and did not necessitate further intervention. Only 7.1% of patients underwent redo surgery within three years, underscoring that anatomical recurrence does not equate to clinical failure. This distinction between radiological findings and symptomatic outcomes is particularly important when counselling patients, as many anatomical recurrences remain small and clinically silent [3,4].

Our results are consistent with large series that demonstrate progressive radiological recurrence but a relatively low requirement for reoperation. Le Page et al., in a cohort of 455 patients, reported increasing recurrence over time but durable quality-of-life improvements and a redo rate of approximately 5% [3]. Similarly, Furtado et al. observed recurrence in about one-quarter of patients at two years, yet overall symptom improvement was preserved [4]. More recent data from Nguyen et al., involving over 800 patients, also confirmed that while 27% developed anatomical recurrence at around three years, fewer than one-third of symptomatic recurrences required surgical revision [5]. Collectively, these findings, alongside our own, indicate that symptom-led follow-up is a more clinically relevant strategy than reliance on radiological surveillance alone.

When recurrence requires redo surgery, outcomes are generally less favourable than after primary repair, with higher morbidity and less predictable symptom resolution. Nevertheless, acceptable results can be achieved in carefully selected patients following thorough evaluation. Narrative reviews emphasise reserving reoperation for those with significant symptomatic recurrence [6]. Another important factor in long-term management is the use of standardised follow-up and reporting. Inconsistencies in defining recurrence-whether purely anatomical or clinically significant-complicate comparisons between studies and highlight the value of incorporating patient-reported outcomes into monitoring strategies [7]. Expert recommendations further support this approach, reinforcing the role of careful patient selection and structured evaluation in guiding redo surgery [8].

The role of mesh reinforcement also remains debated. Some meta-analyses suggest that mesh may reduce overall recurrence, although it does not reliably prevent large recurrences or the need for reoperation, and synthetic mesh carries an erosion risk [9]. These findings support a selective rather than routine approach to mesh use. Our results also reflect the long-term durability patterns described by Ugliono et al., who reported temporal trends in revisional surgery after large hiatal hernia repair [10].

This study has several strengths, including the use of consecutive cases, clear reporting of both anatomical and clinical endpoints, and a complete intention-to-follow approach for all 126 patients. However, limitations must also be acknowledged. The retrospective design introduces potential selection and reporting bias, and validated quality-of-life instruments were not systematically applied. In addition, operative variables such as the type of fundoplication or the use of mesh were not analysed, limiting conclusions about technical factors that may influence recurrence.

Taken together, these results reinforce the role of laparoscopic repair as a safe and effective treatment for symptomatic hiatal hernia, with long-term benefits maintained in most patients. The findings also emphasise the importance of a pragmatic approach to follow-up, in which radiological recurrence alone should not dictate reoperation unless accompanied by significant symptoms.

## Conclusions

Laparoscopic hiatal hernia repair offers reliable long-term symptom control, with most patients maintaining meaningful improvement three years after surgery. Radiological recurrence becomes more frequent over time, but in the majority of cases, it remains asymptomatic and does not require further intervention. Only a small minority of patients proceed to redo surgery, supporting a symptom-focused strategy rather than radiological surveillance alone. These findings emphasise the importance of aligning surgical decision-making with patient-reported outcomes and reinforce the role of laparoscopic repair as a durable and effective treatment in a specialist setting.
